# Targeting Cyclooxygenase-2 in Pheochromocytoma and Paraganglioma: Focus on Genetic Background

**DOI:** 10.3390/cancers11060743

**Published:** 2019-05-28

**Authors:** Martin Ullrich, Susan Richter, Verena Seifert, Sandra Hauser, Bruna Calsina, Ángel M. Martínez-Montes, Marjolein ter Laak, Christian G. Ziegler, Henri Timmers, Graeme Eisenhofer, Mercedes Robledo, Jens Pietzsch

**Affiliations:** 1 Department of Radiopharmaceutical and Chemical Biology, Helmholtz-Zentrum Dresden-Rossendorf, Institute of Radiopharmaceutical Cancer Research, 01328 Dresden, Germany; v.seifert@hzdr.de (V.S.); s.hauser@hzdr.de (S.H.); 2 Institute of Clinical Chemistry and Laboratory Medicine, University Hospital Carl Gustav Carus at the Technische Universität Dresden, 01307 Dresden, Germany; susan.richter@uniklinikum-dresden.de (S.R.); graeme.eisenhofer@uniklinikum-dresden.de (G.E.); 3 Faculty of Medicine Carl Gustav Carus, School of Medicine, Technische Universität Dresden, 01307 Dresden, Germany; 4 Human Cancer Genetics Programme, Hereditary Endocrine Cancer Group, Spanish National Cancer Research Centre, 28029 Madrid, Spain; bcalsina@cnio.es (B.C.); ammontes@cnio.es (Á.M.M.-M.); mrobledo@cnio.es (M.R.); 5 Department of Internal Medicine; Sections of Endocrinology and Vascular Medicine, Radboud University Medical Centre, 6525 GA Nijmegen, The Netherlands; marjolein.terlaak@outlook.com (M.t.L.); henri.timmers@radboudumc.nl (H.T.); 6 Department of Medicine III, University Hospital Carl Gustav Carus at the TU Dresden, 01307 Dresden, Germany; christian.ziegler@uniklinikum-dresden.de; 7 Centro de Investigación Biomédica en Red de Enfermedades Raras, 28029 Madrid, Spain; 8 Faculty of Chemistry and Food Chemistry, School of Science, Technische Universität Dresden, 01069 Dresden, Germany

**Keywords:** VHL, NF1, EPAS1, hypoxia-inducible factor, inflammation, radiosensitization, succinate dehydrogenase, mouse pheochromocytoma cells, immunohistochemistry, fluorescence imaging

## Abstract

Cyclooxygenase 2 (COX-2) is a key enzyme of the tumorigenesis-inflammation interface and can be induced by hypoxia. A pseudohypoxic transcriptional signature characterizes pheochromocytomas and paragangliomas (PPGLs) of the cluster I, mainly represented by tumors with mutations in von Hippel–Lindau (*VHL*), endothelial PAS domain-containing protein 1 (*EPAS1*), or succinate dehydrogenase (*SDH*) subunit genes. The aim of this study was to investigate a possible association between underlying tumor driver mutations and COX-2 in PPGLs. *COX-2* gene expression and immunoreactivity were examined in clinical specimens with documented mutations, as well as in spheroids and allografts derived from mouse pheochromocytoma (MPC) cells. COX-2 in vivo imaging was performed in allograft mice. We observed significantly higher *COX-2* expression in cluster I, especially in *VHL*-mutant PPGLs, however, no specific association between *COX-2* mRNA levels and a hypoxia-related transcriptional signature was found. COX-2 immunoreactivity was present in about 60% of clinical specimens as well as in MPC spheroids and allografts. A selective COX-2 tracer specifically accumulated in MPC allografts. This study demonstrates that, although pseudohypoxia is not the major determinant for high COX-2 levels in PPGLs, COX-2 is a relevant molecular target. This potentially allows for employing selective COX-2 inhibitors as targeted chemotherapeutic agents and radiosensitizers. Moreover, available models are suitable for preclinical testing of these treatments.

## 1. Introduction

Over the past few decades, advances in genetic testing have substantially facilitated the identification of germline and somatic mutations in tumor susceptibility genes in about 60% of adrenal pheochromocytomas and their extra adrenal counterparts, paragangliomas (summarized as PPGLs) [1,2,3,4]. Gain-of-function mutations in proto-oncogenes such as rearranged during transfection (*RET*; germline or somatic), endothelial per-arnt-sim domain-containing protein 1 (*EPAS1*; somatic), and Harvey rat sarcoma viral oncogene homolog (*HRAS*; somatic); and loss-of-function mutations in tumor suppressor genes such as von Hippel–Lindau (*VHL*; germline or somatic), neurofibromin 1 (*NF1*; germline or somatic), transmembrane protein 127 (*TMEM127*; germline), and myc-associated factor X (*MAX*, germline or somatic), as well as mutations in all four succinate dehydrogenase subunits (*SDHD*, *SDHC*, *SDHB*, and *SDHA*; germline) and SDH assembly factor 2 (*SDHAF2*; germline) have been implicated in the tumorigenesis of PPGLs [2,5,6,7,8,9,10,11]. Beyond that, the number of PPGL susceptibility gene candidates is still increasing, e.g. [4,12,13,14,15,16,17,18,19,20,21], although most of them seem to play a minor role in PPGL according to the low proportion of patients related to these genes described so far.

Gene expression profiling provided the basis for classifying PPGLs according to their main transcriptional signatures underlying the aforementioned mutations: cluster I presents with activation of pseudohypoxic signaling pathways and includes mainly *VHL-*, *EPAS1-*, and *SDHx-*mutant cases; cluster II is enriched in kinase receptor signaling pathways and is comprised of *RET-, NF1-*, *TMEM127-*, *MAX-*, and *HRAS-*mutant cases [22,23]. Both pseudohypoxia and kinase receptor signaling are involved in regulating apoptosis, proliferation, invasion, and metastasis, and angiogenesis via different mechanisms, but can also contribute to inflammatory conditions in various tumor entities [24,25,26,27].

Cyclooxygenases (COX) 1 and 2, also referred to as prostaglandin-endoperoxide synthases (PTGS 1 and 2; EC 1.14.99.1), catalyze the conversion of arachidonic acid to prostaglandin H_2_ (PGH_2_). PGH_2_ is then converted into a variety of other prostanoids, determined by certain downstream synthase and isomerase pathways. Prostanoids comprise other prostaglandins such as PGE_2_ and PGF_2_α, prostacyclin (PGI_2_) and thromboxanes (e.g., TXA_2_). These compounds are ligands for G protein-coupled receptors and act as potent para- and endocrine mediators of metabolic processes in homeostasis, but also in inflammatory and neoplastic processes. In particular, the inducible isoenzyme COX-2 is a key enzyme of the tumorigenesis-inflammation interface. In this context, COX-2 overexpression has been shown in various tumor entities and is positively correlated with progression, malignancy and poor patient survival [28]. COX-2 overexpression also contributes to chemo- and radiation resistance [29,30,31,32,33,34,35]. Hypoxic and pseudo-hypoxic signaling additionally influences COX-2-mediated pathways [26,27,36]. Therefore, studies have been initiated on the use of selective COX-2 inhibitors (coxibs) as targeted chemotherapeutic agents and potential radiosensitizers [28,37].

Endoradiotherapy, e.g., with [^177^Lu]Lu-DOTA-(Tyr^3^)octreotate (^177^Lu-DOTA-TATE) is currently investigated as a treatment option for inoperable or metastatic PPGLs, showing promising effects, but sometimes incomplete tumor remission in clinics as well as in preclinical PPGL models [38,39,40]. COX-2 is associated with chemo- and radiation resistance and poor patient outcome in a number of tumor entities [29,30,31,32,33,34,35] encouraging us to investigate whether COX-2 is a potential target in PPGLs.

The first report on COX-2 gene expression and immunohistochemistry in adrenal pheochromocytomas was published in 2001 suggesting that the enzyme might have a role in malignant transformation of these tumors [41]. Between 2007 and 2011, another four immunohistochemical studies were published supporting the value of COX-2 as surrogate marker that, in association with other factors, could potentially discriminate between benign and metastatic pheochromocytoma [42,43,44,45]. Due to literature showing that COX-2 is induced by hypoxia signaling [46,47], we hypothesized that cluster I PPGLs have a higher COX-2 expression than cluster II. Accordingly, COX-2 may be a promising molecular target for functional imaging and adjuvant treatment, in particular in cluster I PPGLs.

To address the above hypothesis, we evaluated COX-2 status of PPGLs with known mutational status for *VHL*, *SDHx*, *EPAS1*, *NF1*, *RET*, and *HRAS* on both mRNA and protein level. Furthermore, we characterized COX-2 immunoreactivity in tumor spheroids and allografts derived from mouse pheochromocytoma (MPC) cells with a heterozygous *Nf1* knockout [48,49] in order to assess the usefulness of these models for preclinical testing of COX-2-targeting adjuvant and, in particular, radiosensitizing treatments.

## 2. Results

### 2.1. COX-2 Gene Expression in Clinical PPGL Samples

*COX-2* gene expression data were extracted from gene expression arrays [50,51] of 70 PPGL samples with documented mutations in tumor susceptibility genes (Table 1). This series reflects the expected age, location, and metastatic disease, according to the mutations involved. Most cases were adrenal pheochromocytomas (67%), followed by thoracic and abdominal paragangliomas (29%), and head and neck paragangliomas (7.1%). Germline mutations were documented in 60% of cases. At the time of investigation 8.6% of cases showed metastatic disease. Most of the tumors carried mutations in *SDHx* (23% comprising 5 *SDHD*, 2 *SDHC*, and 9 *SDHB,* cases) and *RET* (23%) followed by *VHL* (21%), *EPAS1* (16%), *HRAS* (11%), and *NF1* (5.7%). The *SDHx* subgroup showed the highest proportion of extra-adrenal paragangliomas (62% thoracic and abdominal, and 31% head and neck), followed by *EPAS1* (73% thoracic and abdominal). All other genetic subgroups included mostly adrenal pheochromocytomas (85−100%). All subgroups showed similar means in tumor diameters (4.4−5.9 cm). Metastatic disease was most frequently documented among *SDHx*-mutant cases (25%) compared to all other genetic subgroups (0−18%).

Statistical analysis taking into account the general clinico-morphologic features of the tumors showed that gender, tumor location, tumor diameter, age at diagnosis, or metastatic behavior had no relevant influences on *COX-2* gene expression (Table S1). On the other hand, *COX-2* expression was significantly higher in head and neck PPGLs *(p* = 0.01) compared to other locations. Of note, all head and neck PPGLs in this series were related to an *SDHD* germline mutation.

**Figure 1:**
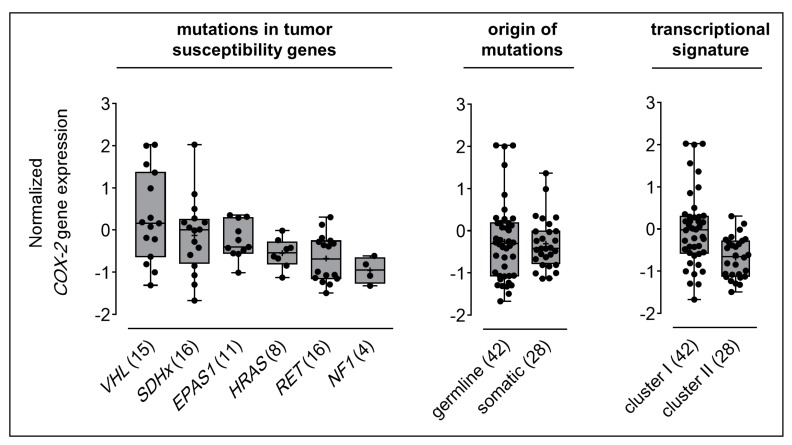
Normalized *COX-2* gene expression in PPGLs with regard to genetic background. gene expression array data were derived from Lopez-Jimenez et al. [50] and Qin et al. [51] mRNA expression series. Seventy samples with known genotype were included in this analysis and classified according to the specific gene mutated, origin of mutations, and transcriptional cluster; numbers in parentheses represent the number of samples investigated in each subgroup; see Table S1 for statistical analyses.

PPGLs carrying a *VHL* mutation showed the highest *COX-2* expression (0.30 ± 0.28), followed by cases with *SDHx* (−0.13 ± 0.22), *EPAS1* (−0.25 ± 0.13), *HRAS* (−0.55 ± 0.12), *RET* (−0.68 ± 0.14), and *NF1* (−0.96 ± 0.16) mutations (Figure 1). The mean *COX-2* expression among all cluster I tumors was higher (−0.01 ± 0.14) compared to cluster II (−0.68 ± 0.09). *COX-2* expression was similar in hereditary (−0.26 ± 0.15) and somatic cases (−0.31 ± 0.11). *COX-2* expression showed significant positive relationships with *VHL* mutations (*r* = 0.371, *p* = 0.002) and cluster I transcriptional signature (*r* = 0.406, *p* < 0.001), respectively.

To further investigate a possible association between the pseudohypoxic signature and *COX-2* in cluster I PPGLs, unsupervised clustering for 97 hypoxia-related genes (see materials and methods for details) showed that *VHL-* and *SDHx*-mutant cases clustered together (Figure S1). Pearson correlation for *COX-2* expression and pseudohypoxic signature indicated significant relationships (*p* < 0.05) for 86 out of 171 probes, representing 65 out of 97 genes related to hypoxia in PPGLs (Table S2). Correlation coefficients ranged from 0.19 to 0.63 with 65 probes having only a weak correlation below 0.4. Exemplary, consistent with high *COX‑2* expression, the analysis showed a significant positive relationship with mRNA levels of Ca^2+^-dependent phospholipase A2 (*r* = 0.564, *p* < 0.001) since the enzyme is required for releasing arachidonic acid from phospholipid membranes as the specific substrate for cyclooxygenases.

### 2.2. COX-2 Immunoreactivity in Clinical PPGL Tissue Samples of a Second Cohort

COX-2 immunoreactivity was assessed in formalin-fixed paraffin-embedded tumor samples from a separate cohort sharing no case with the RNA sample cohort. This series included 96 PPGLs with a clinically documented mutation in tumor susceptibility genes (Table 2) and reflects the expected age, location, and metastatic disease, according to the mutations involved. Most cases were adrenal pheochromocytomas (52%), followed by thoracic and abdominal paragangliomas (26%), and head and neck paragangliomas (22%). Germline mutations were documented in 52% of cases. At the time of investigation, 13% of cases showed metastatic disease.

Most of the cases carried a mutation in *SDHx* (41% comprising 18 *SDHD*, 3 *SDHC*, 9 *SDHB,* 5 *SDHA*, and 4 *SDHAF2* cases) followed by *NF1* (22%), *VHL* (15%), *RET* (9.4%), *EPAS1* (7.3%), and *HRAS* (6.3%). The *SDHx* subgroup showed the highest proportion of extra-adrenal paragangliomas (39% thoracic and abdominal, and 51% head and neck), whereas all other genetic subgroups included mostly adrenal pheochromocytomas (57−95%). Tumor diameters were significantly smaller in the *SDHx* subgroup (3.9 cm) and significantly higher in the *RET* subgroup (7.2 cm) compared to cases with other genetic backgrounds. Metastatic disease was most frequently documented among *SDHx* cases (21%) compared to all other genetic subgroups (0−17%).

**Figure 2:**
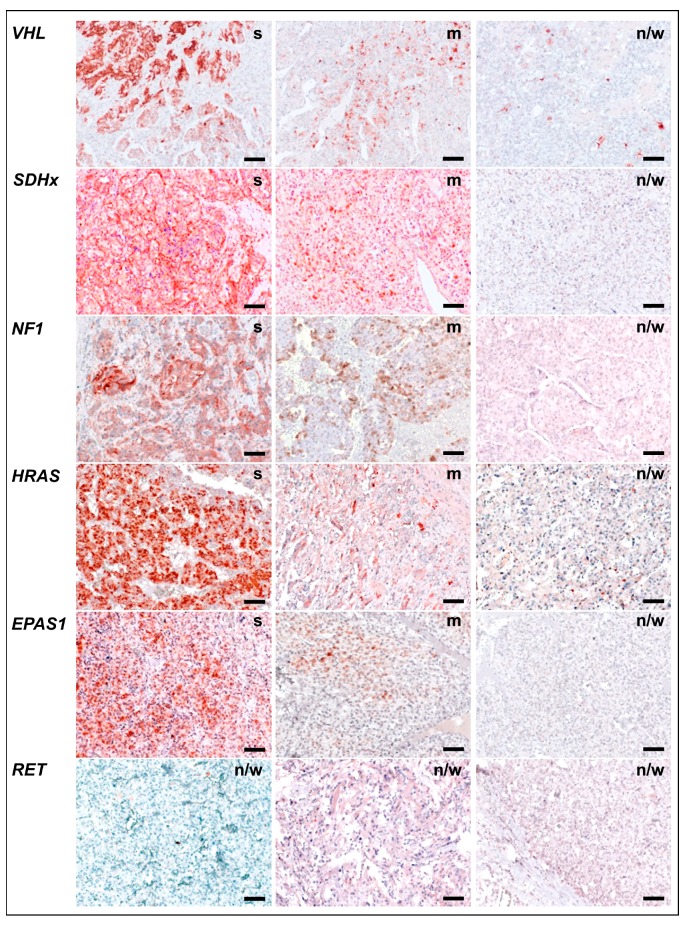
Scoring of COX-2 immunoreactivity in PPGL tissue samples; examples for cases carrying loss-of-function-mutations in *VHL*, *SDHx*, or *NF1*, or gain-of-function mutations in *HRAS*, *EPAS1*, or *RET*, (s) strong immunoreactivity, >50% of tumor cells were stained; (m) moderate immunoreactivity, 20–50% of tumor cells were stained; (n/w) negative or weak, <20% of tumor cells were stained; scale bars: 0.1 mm.

COX-2 immunoreactivity was assessed by three observers using a three-mark score taking into account the percentage of positively stained tumor cells per tissue section (Figure 2). Sections with ‘strong’ (>50% of tumor cells stained) or ‘moderate’ score (20–50% of tumor cells stained) showed cytoplasmic COX-2 immunoreactivity in pheochromocytes and/or interconnected stromal cells. Tumors with ‘negative or weak’ score (<20% of tumor cells stained) showed COX-2 immunoreactivity predominantly in few stromal cells scatted over the tissue section. Interobserver variation statistics showed good agreement between the first two observers (weighted κ = 0.67). However, there were 14 cases of disagreement (15%) between ‘negative or weak’ and ‘moderate’ scores as well as 16 cases of disagreement (17%) between ‘moderate’ and ‘strong’ scores that where passed to a third observer for final decision.

COX-2 immunoreactivity was strong in 23 samples (24%) and moderate in 35 samples (36%) whereas 38 samples (40%) showed negative or weak staining. Tumor location, tumor diameter, age at diagnosis, or metastatic behavior had no statistically relevant influences on COX-2 immunoreactivity (Table S1). On the other hand, COX-2 immunoreactivity was significantly higher in samples from male patients compared to females and all genetic subgroups showed different sex ratios. However, multiple regression analyses, testing the relationships between COX-2 expression and the two independent variables ‘genetic background’ and ‘sex’ simultaneously, showed that trends in COX-2 immunoreactivity of the genetic subgroups were not artifacts of different sex ratios.

**Figure 3:**
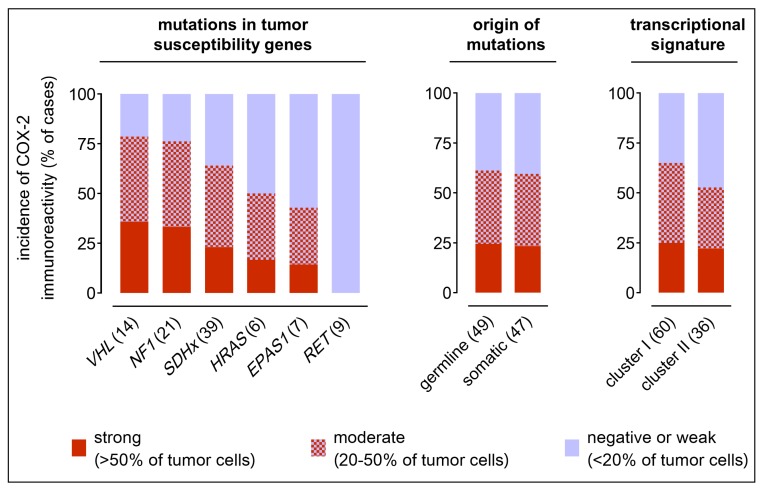
Comparison of COX-2 immunoreactivity in PPGLs in respect to genetic background; incidences of strong, moderate, and negative or weak COX-2 immunoreactivity observed among 96 tissue samples classified with regard to specific mutations in tumor susceptibility genes, origin of mutations, and transcriptional cluster; numbers in parentheses represent the number of samples investigated in each subgroup; see Table S1 for statistical analyses.

In tissue samples with different genetic backgrounds, a trend was observed with highest COX-2 immunoreactivity in PPGLs due to *VHL* mutations (36% strong, 43% moderate), followed by *NF1* (33% strong, 43% moderate), *SDHx* (23% strong, 41% moderate), *HRAS* (17% strong, 33% moderate), *EPAS1* (14% strong, 29% moderate), and RET (all samples negative or weak) (Figure 3). Of note, COX-2 immunoreactivity showed similar incidences in different *SDH* subtypes. However, due to higher numbers of *SDHD*-mutant cases compared to the other subtypes, multiple regression analyses (Table S2) taking also into account the sex of the patients showed a significant positive relationship between *SDHD* mutation and COX-2 immunoreactivity (*r* = 0.867, *p* ≤ 0.001). A negative relationship was detected between *RET* mutation and COX-2 immunoreactivity (*r* = −0.948; *p* < 0.001). COX-2 immunoreactivity was similar in hereditary cases (24% strong, 37% moderate) compared to somatic cases (24% strong, 37% moderate). The trend for higher COX-2 immunoreactivity in cluster I (25% strong, 40% moderate) compared to cluster II (22% strong, 31% moderate) was related to different sex ratios in these groups (*r* = 0.323, *p* = 0.043).

**Figure 4:**
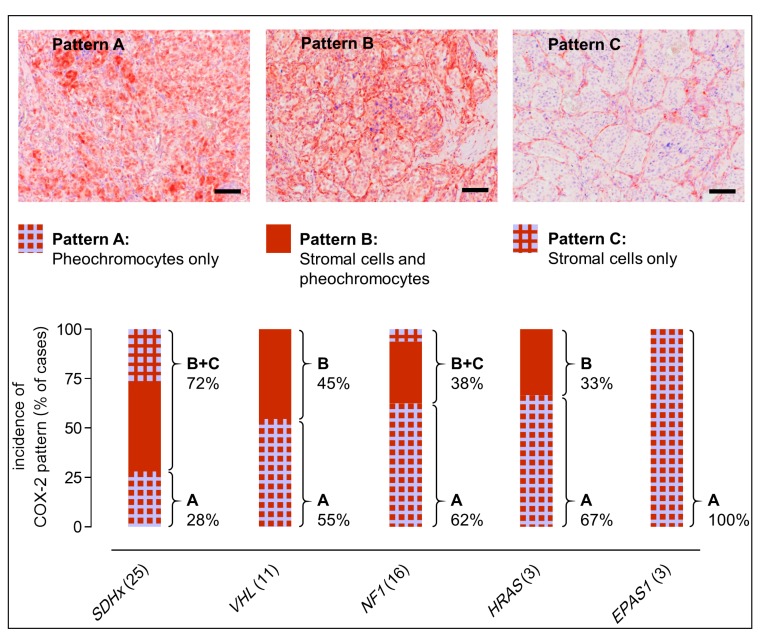
Immunoreactivity pattern in COX-2-positive PPGLs; COX-2 immunoreactivity with stromal cells involved was more frequently observed in tumors related to *SDHx* mutations (72%, pattern B+C) compared to other genetic background (0−45%); histologic examples: (pattern A) *HRAS* somatic mutation; (pattern B) *SDHD* germline mutation; (pattern C) *SDHD* germline mutation; see Table S1 for statistical analyses; scale bars: 0.1 mm.

All 58 COX-2-positive PPGLs were further stratified in terms of their histologic staining pattern (Figure 4). Three different patterns of COX-2 immunoreactivity were observed: (pattern A) staining of pheochromocytes only, (pattern B) staining of both stromal cells and pheochromocytes, and (pattern C) staining of stromal cells only. Tumors with mutations in *SDHx* showed the highest proportion of COX-2 immunoreactivity with stromal cells involved (72%, pattern B+C), followed by *VHL* (45%, pattern B only), *NF1* (38%, pattern B+C), *HRAS* (33%, pattern B only), and *EPAS1* (0%). Pearson correlation showed a significant positive relationship between *SDHx* mutations and COX-2 immunoreactivity with stromal cells involved (*r* = 0.266, *p* = 0.009).

### 2.3. COX-2 as Molecular Target in Preclinical PPGL Models

**Figure 5:**
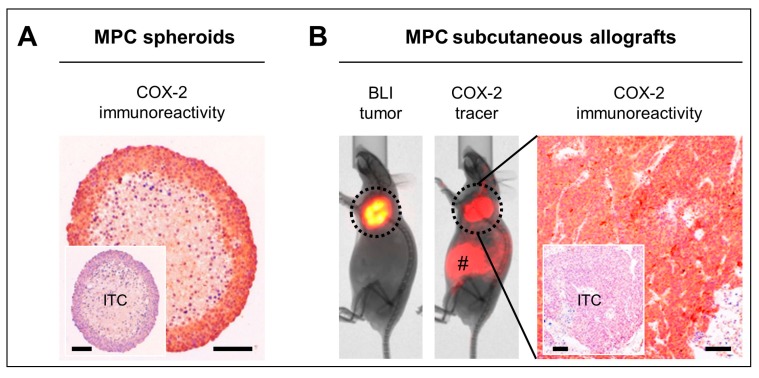
COX-2 as a molecular target in MPC spheroids and allografts harboring a heterozygous *Nf1* knockout; (**A**) COX-2 immunoreactivity in MPC spheroids; (**B**) COX-2 functional imaging and immunoreactivity in a luciferase-expressing subcutaneous MPC allograft model; (BLI) bioluminescence imaging of tumors; (COX-2 tracer) tumor uptake of a red-fluorescent COX-2 selective imaging probe; (#) residual non-specific accumulation of COX-2 tracer due to intraperitoneal injection; (ITC) IgG isotype control; scale bars: 0.1 mm

We further assessed the COX-2 status of a commonly used preclinical model of mouse pheochromocytoma (MPC) cells with heterozygous *Nf1* knockout. In vitro, MPC spheroids showed strong and homogeneous COX-2 immunoreactivity in pheochromocytes involving the most peripheral 8−10 cellular layers, whereas COX-2 immunoreactivity was absent in the necrotic core (Figure 5A). In vivo, MPC tumors in a subcutaneous allograft model showed strong tumor-specific uptake of a red-fluorescent COX-2 imaging probe (Figure 5B). Tissue sections from these allografts showed strong and homogenous COX-2 immunoreactivity predominantly involving pheochromocytes.

## 3. Discussion

Endoradiotherapy, e.g., with ^177^Lu-DOTA-TATE is currently investigated as a treatment option for inoperable or metastatic PPGLs, showing promising effects, but sometimes incomplete tumor remission in clinics as well as in preclinical models [38,39,40]. COX-2 is associated with chemo- and radiation resistance and poor patient outcome in a number of tumor entities [29,30,31,32,33,34,35] encouraging us to investigate whether COX-2 is a potential target in PPGLs. Inhibition of COX-2 is considered a viable radiosensitization strategy [24,35]. In particular, selective COX-2 inhibitors (coxibs) have been suggested for combination radiotherapy of tumors, thereby enhancing radiosensitivity in various settings [28,35,37].

Expression of COX-2 was assessed on mRNA or protein level in two separate cohorts of PPGL patients with known tumor driver mutations. Both cohorts were comprised of tumors with a similar distribution of clinical features in respect to sex, tumor location, age at diagnosis, and metastatic behavior, reflecting previously described features of PPGLs [52].

Despite a significant increase in *COX-2* mRNA levels in cluster I compared to cluster II PPGLs, we did not find a significant relationship between the pseudohypoxic transcriptional signature of cluster I PPGLs and *COX-2* in clinical samples. COX-2 protein levels are consistent with these results showing also no significant difference between cluster I and cluster II PPGLs. This may be due to the fact that PPGLs are characterized by a high degree of intertumoral heterogeneity. Many different factors have been described to activate and interfere with COX-2 in cancer [28]. Amongst others, intratumoral differences in normoxic/hypoxic conditions, systemic chemotherapy, oxidative stress, or even tobacco smoking can interfere with COX-2 levels on gene expression and protein level, possibly masking a potential association with the pseudohypoxic signature of cluster I PPGLs. Nevertheless, trends were observed that may at least partially be explained by the underlying genetic background.

In cluster I PPGLs with loss-of-function mutations in *VHL* and *SDHx*, pseudohypoxic transcriptional phenotypes may contribute to COX-2 induction on gene expression and protein level. Functional defects of VHL, an E3 ubiquitin ligase, directly impair ubiquitin labeling of hypoxia-inducible factors (HIF-α) for regular proteasomal degradation. Functional defects of SDH indirectly impair ubiquitin-labeling of HIF-α caused by intracellular accumulation of succinate, an intrinsic inhibitor of prolyl hydroxylases. Therefore, both VHL and SDH defects are associated with enhanced HIF-α signaling even under normoxic conditions, a metabolic state referred to as pseudohypoxia [25]. From investigations on other tumor entities, in particular on colon cancer, it is known that both HIF-α isoforms (1 and 2) are capable of directly upregulating *COX-2* expression [26,27].

*COX-2* mRNA levels as well as the percentage of moderate and high COX-2 immunoreactivity tended to be lower among cases carrying a gain-of function mutation in *EPAS1*, encoding the HIF-2α protein, compared to *VHL-* and *SDHx*-mutant cases. This observation suggests that activation of HIF-2α alone may not be sufficient for COX-2 upregulation in PPGLs.

In cluster II PPGLs with loss-of-function mutations in *NF1* gene, the trend for a relatively high COX-2 immunoreactivity is consistent with another report on elevated COX-2 and prostaglandin E2 (PGE_2_) levels in *NF1* malignant peripheral nerve sheath tumors [53]. These observations raise the possibility that functional defects in NF1, a GTPase-activating protein, could indirectly contribute to the upregulation of *COX-2* expression. This may at least be partly explained by elevated levels of activated Ras-GTP leading to hyperactivation of mitogen-activated protein kinase (MAPK) pathways. Under normoxic conditions, *COX-*2 expression can be induced through activation of oncogenic pathways such as Ras-MAPK and can even be further enhanced by HIF-1α during hypoxia [36]. The lack of high *COX-2* mRNA in the gene expression cohort might be due to the low number of *NF1*-mutant cases in this particular set of tumors. On the other hand, all *NF1* cases in the RNA sample cohort carried germline mutations, whereas all *NF1* cases in the tissue sample cohort carried somatic mutations. Whether there is a relationship between germ line or somatic *NF1* mutations and different COX-2 levels in PPGLs remains to be investigated.

In accordance with the observations in clinical PPGL samples, COX-2 immunoreactivity was also high in spheroids and subcutaneous allografts derived from mouse pheochromocytoma (MPC) cells with a heterozygous *Nf1* knockout. Therefore, these models are suitable for preclinical testing of COX-2-targeted treatments for the management of PPGLs. Since we did not find a significant relationship between tumor driver mutations and COX-2 in clinical PPGL samples, molecular imaging could be applied in a personalized approach to pre-estimate whether a tumor is susceptible to COX-2-targeted treatment. In our study, specific accumulation of a red-fluorescent COX-2 probe in subcutaneous MPC allografts demonstrates the potential value of COX-2 tracers for assessing the target status in PPGLs non-invasively. In order to translate this approach into clinical practice, studies have been initiated on the use of selective COX-2 inhibitors as PET radiotracers for cancer imaging [54,55,56].

In the case of HRAS and RET mutations, trends for lower *COX-2* mRNA levels in PPGLs are in agreement with COX-2 immunoreactivity. These findings suggest that both *HRAS* and *RET* have not major role in regulating *COX-2* expression in PPGLs. However, there have been reports on fibroblasts transformed with a mutant *HRAS* responding with a rapid induction of *COX-2* on gene expression and protein level [57]. It has also been reported that *HRAS* expression increases *COX-2* expression in intestinal epithelial cells [58]. In thyroid cancer, RET has been shown to activate Ras, and thus it could indirectly lead to *COX-2* activation, however, whether RET could activate *COX-2* in any other way is a matter of investigation [59].

The observation that tumor diameter and age at diagnosis had no statistically relevant impact on COX-2 levels is in accordance with previous studies [41,42,43,44,45]. In contrast to these reports, we did not detect a statistically relevant increase of *COX-2* mRNA and COX-2 immunoreactivity in primary tumors of metastatic PPGLs. This is most likely due to the relatively small number of metastatic cases in our cohorts. Significantly higher *COX-2* mRNA levels in head and neck PPGLs compared to other tumor locations is related to the fact that all head and neck PPGLs in this series carried an *SDHD* mutation. This raises the possibility that COX-2 expression may be regulated by *SDHD*-related metabolic alterations in particular in head & neck PPGLs. However, due to low sample numbers available in the *SDHD* subgroup, further studies focusing on COX-2 expression in specific *SDH* mutation subtypes are necessary to draw a conclusion from these initial observations. In our tissue series, head and neck PPGLs comprised of cases with different tumor driver mutations explaining why similar effects on COX-2 were not detected in this series. Significantly higher COX-2 protein in PPGLs from male patients compared to females is considered a specific characteristic of our tissue sample cohort since we did not detect a similar effect in the RNA samples.

Higher COX-2 immunoreactivity in stromal cells of PPGLs related to *SDHx* germline mutations compared to other tumor driver mutations indicates a systemic effect of partial *SDHx* loss on COX-2 levels. This pattern of COX-2 immunoreactivity may be related to structure-supporting sustentacular cells and/or a characteristic monocytic component in PPGLs that has recently been discovered [60]. Further histologic investigations are required to elucidate the specific cell populations of stromal COX-2 immunoreactivity in PPGLs. A report on COX-2 in cervical cancer showed that the ratio between COX-2 in tumor cells and COX-2 in stroma cells was very effective in distinguishing patients with low versus high risk of death from disease. A very strong relationship between both tumor *COX-2* expression and tumor-to-stromal COX-2 ratio has been shown to be highly correlated with response to chemotherapy while, high *COX-2* expression in the stroma was significantly associated with better survival, but failed to directly correlate with response to treatment [61]. Further studies are required to fully elucidate the role of COX-2 in PPGL tumorigenesis and therapy resistance.

## 4. Materials and Methods

### 4.1. Tumor Samples and Genetic Testing

For immunohistochemical analysis, a series of 96 tumors from patients with confirmed PPGL diagnosis were used for this study. The cohort was recruited in the four participating centers: Tumor and Normal Tissue Bank of the UCC/NCT at the Universal Hospital Carl Gustav Carus, Dresden, Germany, Spanish National Cancer Research Centre, Madrid, Spain, Radboud University Medical Centre, Nijmegen, The Netherlands, and University of Florence, Italy. All patients provided informed consent to collect clinical and genetic data, in accordance with institutional ethical-approved protocols for each center. Metastatic cases were defined based on clinical documentation of metastases or extensive local invasion. Genetic screening was performed in germline and tumor DNA using a next-generation sequencing panel (PheoSeq) as previously described [62].

### 4.2. Gene Expression Profiling and Data Processing

Gene expression array data were extracted from [50,51]. To investigate the association between a pseudohypoxic transcriptional signature and *COX-2* in cluster I PPGLs on RNA level, a published list of 782 genes significantly differentially expressed between *VHL*- and *SDHB*-mutant cases was taken into account [50]. These genes were compared with the hypoxia database including all genes theoretically related to hypoxia [63]. Unsupervised clustering was applied with 97 hypoxia-related genes overlapping from both lists (Figure S1). Pearson correlation coefficients^®^ were calculated between *COX-2* expression and each of the 97 genes.

### 4.3. COX-2 Immunohistochemistry

Formalin-fixed and paraffin-embedded tumor and spheroid sections (3 µm) were dewaxed using Roticlear (Carl Roth, Karlsruhe, Germany) and rehydrated in a graded series of ethanol. Antigen retrieval was performed in 10 mmol/L citrate buffer pH 6 intermittently heated to 100 °C in 5 min intervals. Washing was performed using 0.05 mol/L Tris-buffered saline pH 8 containing 0.5% (v/v) Tween-20 (TBS-T). Endogenous peroxidase was quenched using 3% H_2_O_2_ in TBS-T. Endogenous avidin and biotin were blocked using a commercially available avidin/biotin quenching system (Agilent, Santa Clara, CA, USA). Non-specific binding sites were blocked using 10% fetal bovine serum (v/v) in TBS-T. COX-2 was detected using the primary antibody ab15191 (Abcam, Cambridge, UK). Isotype controls were incubated with non-specific rabbit IgG ab27478 (Abcam). Specific binding was detected using the biotinylated secondary antibody 111-065-003 (Dianova, Hamburg, Germany) and ExtrAvidin-peroxidase E2886 (Sigma-Aldrich, St. Louis, MO, USA) followed by staining with *3*-amino-*9*-ethylcarbazole substrate (Sigma-Aldrich). Tumor sections were counterstained with Meyer’s hematoxylin, mounted with Kaiser’s glycerol gelatin (Carl Roth), and imaged using the AXIO Imager A1 microscope (Carl Zeiss, Oberkochen, Germany).

### 4.4. Scoring of COX-2 Immunoreactivity

For each case, COX-2 immunoreactivity was analyzed from a series of bright field images (magnification, ×100) contiguously captured along the diameter of one tumor section (images per section > 5). Perinuclear and cytoplasmic red-brown staining was considered positive. PPGLs form dense, reticular to glandular ‘zellballen’ or intermediate forms [64]. Typically, structure-supporting sustentacular cells are closely associated with tumor cells [65]. Taking into account these specific histologic features of PPGLs, our examination assessed COX-2 immunoreactivity in both inflammatory and sustentacular cells of the stromal compartment and/or pheochromocytes.

The percentage of COX-2-positive tumor cells was assessed using a three-mark score adapted from [41]: negative or weak (<20% of tumor cells); moderate (20–50% of tumor cells); strong (>50% of tumor cells). Samples were evaluated independently by two histologically experienced observers (Martin Ullrich and Verena Seifert) who were blinded to the genetic subtype of tumors. In cases of disagreement, samples were referred to a third observer for final decision (Sandra Hauser). Notably, our scoring system does not report on staining intensities that were observed to vary between samples from different centers most likely due to differences in tissue quality, preservation techniques, and storage time. 

### 4.5. Spheroid Models

Mouse pheochromocytoma cells (MPC clone 4/30PRR [48]) were cultivated as previously described [49]. Spheroids were generated from MPC cells passage 34 as described elsewhere [66,67]. After 18 days of cultivation, spheroids (diameters between 500 and 600 µm) were fixed in paraformaldehyde and embedded in paraffin according to standard procedures (*n* = 6).

### 4.6. Tumor Allograft Models

Animal experiments were carried out at the Helmholtz-Zentrum Dresden-Rossendorf according to the guidelines of German Regulations for Animal Welfare and have been approved by the local Animal Ethics Committee for Animal Experiments (Landesdirektion Dresden, Germany). Subcutaneous tumor allografts were generated through injection of luciferase-expressing MPC^LUC/eGFP-ZEO^ cells (abbreviated as MPC^LUC/GZ^) passage 11 into female NMRI-nude mice (Rj:NMRI-*Foxn1^nu^*, homozygous, T cell-deficient, hairless; Janvier Labs, Le Genest-Saint-Isle, France) as described previously. Five weeks after cell injection, optical in vivo imaging was performed (tumor diameters between 0.8 and 1.2 mm). After imaging, mice were sacrificed using CO_2_ inhalation and cervical dislocation. Tumors were excised, fixed in paraformaldehyde, and embedded in paraffin according to standard procedures (*n* = 6).

### 4.7. Optical In Vivo Imaging

Optical tumor imaging in mice was performed on a preclinical In-Vivo Xtreme imaging system (Bruker, Billerica, MA, USA) under general anesthesia with inhalation of 10% (v/v) desflurane (Baxter, Unterschleißheim, Germany) in 30% (v/v) oxygen air. Location and morphology of luciferase-expressing MPC^LUC/GZ^ allografts were assessed using bioluminescence imaging (BLI) as described previously [49]. Functional COX-2 imaging was performed using the RediJect COX-2 Fluorescent Imaging Probe (PerkinElmer, Waltham, MA, USA) injected intraperitoneally according to manufacturer’s instructions. Fluorescence imaging (FLI) was performed three hours after injection. Specific fluorescence of the imaging probe was captured at *λ*_Ex/Em_ = 570/600 nm and non-specific fluorescence was captured at *λ*_Ex/Em_ = 480/535 nm. Tumor uptake was analyzed in processed images, showing specific fluorescence/non-specific fluorescence ratios.

### 4.8. Statistical Analysis

Graphs were drawn using Prism version 5.02 (GraphPad Software, San Diego, CA, USA). Incidences of COX-2 immunoreactivity within a defined subgroup are presented as percent of cases, *n* represents the number of cases. If not stated differently, data are presented as means ± standard error of the means. Significance of differences was tested for *n* ≥ 6 using the Mann–Whitney *U* test. Multiple linear regression analysis was performed using OriginPro 2017G (OriginLab Corporation, Northhampton, MA, USA). Relationships were described with the regression coefficient *r* and considered significant at *p*-values < 0.05. Interobserver variation was calculated using online QuickCalcs κ statistics version 06/2014 (GraphPad Software, www.graphpad.com/quickcalcs/kappa1).

## 5. Conclusions

Moderate to high cyclooxygenase 2 (COX-2) gene expression and immunoreactivity in about 60% of PPGLs demonstrates that, for these patients, COX-2 is considered a clinically relevant molecular target for adjuvant, in particular radiosensitizing treatments using selective COX-2 inhibitors, e.g., in combination with ^177^Lu-DOTA-TATE endoradiotherapy. However, taking into account the genetic background of the samples, is an indicator but not the major determinant for *COX-2* expression in PPGLs. High COX-2 immunoreactivity in tumor spheroids and subcutaneous tumor allografts derived from mouse pheochromocytoma (MPC) cells demonstrates that available PPGL models are suitable for preclinical in vitro and in vivo testing of COX-2-targeting treatments.

## Figures and Tables

**Table 1:** Tumor characteristics and clinical features of 70 PPGL patients extracted from [50,51] for *COX-2* gene expression analysis; (A) adrenal; (TA) thoracic and abdominal; (HN) head and neck.

Mutant Gene	*VHL*	*SDHx ^1^*	*EPAS1*	*NF1*	*RET*	*HRAS*	Total
Total cases (*n*)	15	16	11	4	16	8	70
Hereditary (*n*)	10	15	0	3	14	0	42
Sex (*n*)							
Female	6	9	10	4	8	3	40
Male	9	7	1	0	8	2	27
Unknown	0	0	0	0	0	3	3
Tumor location (*n*)							
A	13	1	5	4	16	8	47
A + TA	2	2	2	0	0	0	6
TA	0	8	4	0	0	0	14
HN	0	5	0	0	0	0	5
Tumor diameter (*n*)							
<4 cm	1	6	3	0	4	0	14
≥ 4 and ≤ 8 cm	4	3	5	0	1	4	17
>8 cm	0	1	2	0	1	1	5
Unknown	10	6	1	4	10	3	34
Mean (cm)	4.4 ± 1.0	4.6±1.2	5.8 ± 1.2	n.a.	4.6 ± 1.2	5.9 ± 0.9	5.1 ± 0.5
Age at diagnosis (years)							
Range	9−47	10−95	18−78	38−58	18−62	45−79	9−97
Unknown	1	0	0	1	0	2	4
Mean	24 ± 3.1 ^†^	27 ± 7.9	42 ± 6.4	48 ± 5.8	38 ± 6.5	64 ± 4.6 ^‡^	36 ± 2.3
Metastatic (*n*)	0	4	2	0	0	0	6

^1^ comprising 5 *SDHD*, 2 *SDHC*, and 9 *SDHB* cases; significance of differences tested with Mann–Whitney *U* test: ^†^ *p* < 0.01, ^‡^ *p* < 0.001; (n.a.) not available.

**Table 2:** Tumor characteristics and clinical features of 96 PPGL tissue samples available for COX-2 immunohistochemistry classified with regard to mutations in different tumor susceptibility genes; (A) adrenal; (TA) thoracic and abdominal; (HN) head and neck.

Mutant Gene	*VHL*	*SDHx ^1^*	*EPAS1*	*NF1*	*RET*	*HRAS*	Total
Total cases (*n*)	14	39	7	21	9	6	96
Hereditary (*n*)	7	38	0	0	4	0	50
Sex (*n*)							
Female	6	18	6	9	4	4	47
Male	8	21	1	12	5	2	49
Tumor location (*n*)							
A	10	4	4	20	8	4	50
TA	3	15	3	1	1	2	25
HN	1	20	0	0	0	0	21
Tumor diameter (*n*)							
<4 cm	3	22	2	6	2	2	35
≥4 and ≤8 cm	7	13	5	11	3	3	44
>8 cm	0	2	0	0	3	0	5
Unknown	4	2	0	4	1	0	12
Mean (cm)	4.6 ± 0.6	3.9 ± 0.4 *	4.5 ± 0.6	4.1 ± 0.3	7.2 ± 1.5 *	3.8 ± 0.5	4.4 ± 0.3
Age at diagnosis (years)							
Range	9−49	14−71	17−75	20−74	33−72	28−81	9−81
Mean	25 ± 3.9 ^‡^	38 ± 2.7 ^†^	42 ± 8.7	52 ± 2.8 ^†^	49 ± 4.2	58 ± 7.2 *	42 ± 1.7
Metastatic (*n*)	1	8	0	1	1	1	12

^1^ comprising 18 *SDHD*, 3 *SDHC*, 9 *SDHB,* 5 *SDHA*, and 4 *SDHAF2* cases; significance of differences tested with Mann–Whitney *U* test: * *p* < 0.05, ^†^ *p* < 0.01, ^‡^ *p* < 0.001.

## Supplementary Materials

The following are available online at https://www.mdpi.com/2072-6694/11/6/743/s1, Figure S1: Unsupervised clustering for 97 hypoxia-related genes in PPGLs applied to our mRNA expression series extracted from [50,51]; *VHL-* and *SDHx*-mutant cases clustered together whereas, other cases showed no homogeneous profile, Table S1: Statistical analyses of COX-2 status with regard to clinical characteristics and genetic background of tumors in two independent series of PPGL samples; data in parentheses were calculated from low sample numbers (*n* < 7); as Mann–Whitney U test showed significant sex-related differences in COX-2 immunoreactivity in tissue samples, multiple regression analyses was applied to distinguish whether the trends observed in the genetic subgroups were related to genetic background or different sex ratios only; levels of significance: * *p* < 0.05; † *p* <0.01, ‡ *p* < 0.001, Table S2: Pearson correlation between expression of *COX-2* and 97 hypoxia-related genes in PPGLs in the mRNA expression series extracted from [50,51].
