# Supplementary material for: Targeting Cyclooxygenase-2 in Pheochromocytoma and Paraganglioma: Focus on Genetic Background
Source: Cancers (Basel). 2019 May 28;11(6):743. doi: 10.3390/cancers11060743 (PMC6627450; doi:10.3390/cancers11060743)
Supplement: Supplementary file 1 [file cancers-11-00743-s001.zip › Table_S1-Statistical_analyses_COX-2_vs_clinical_data#02.xlsx.pdf]

Table S 1: Statistical analyses of COX-2 status with regard to clinical characteristics and genetic background of tumors in two independent series of PPGL samples; data in parentheses were calculated from low sample numbers (*n* < 7); as Mann-Whitney *U* test showed significant sex-related differences in COX-2 immunoreactivity in tissue samples, multiple regression analyses was applied to distinguish whether the trends observed in the genetic subgroups were related to genetic background or different sex ratios only; levels of significance: * *P* < 0.05; † *P* <0.01, ‡ *P* < 0.001

**COX-2 expression levels in RNA samples**			
Mann-Whitney U tests			*P* value
Sex	male *vs.* female		0.455
Malignancy	benign *vs.* metastatic		0.825
Tumor location	adrenal *vs.* others		0.486
	(adrenal + thoracic & abdominal) *vs.* others		(0.847)
	thoracic & abdominal *vs.* others		0.767
	(head & neck) *vs.* others		(0.010*)
Pearson correlation		*r*	*P* value
Age at diagnosis		−0.2119	0.088
Tumor diameter		−0.2201	0.197
Mutation	*VHL*	0.371	0.002†
	SDHx	0.098	0.419
	SDHB	−0.145	0.232
	(SDHD)	(0.252)	(0.035*)
	EPAS1	0.015	0.903
	(NF1)	(−0.207)	(0.086)
	RET	−0.267	0.025
	HRAS	−0.121	0.318
Transcriptional signature	cluster I	0.406	< 0.001‡
Origin of mutations	germline	0.03	0.804
**COX-2 immunoreactivity in tissue samples**			
Mann-Whitney U tests			*P* value
Sex	male *vs.* female		0.030*
Malignancy	benign *vs.* metastatic		0.775
Tumor location	adrenal *vs.* others		0.587
	thoracic & abdominal *vs.* others		0.673
	head & neck *vs.* others		0.273
Pearson correlation		*r*	*P* value
Age at diagnosis		−0.1606	0.124
Tumor diameter		−0.1677	0.127
Multiple linear regression considering sex of the patients		*r*	*P* value
Mutation	*VHL* ; male	0.328; 0.307	0.144; 0.053
	SDHx ; male	0.032; 0.318	0.842; 0.048*
	(SDHA) ; male	(0.135); 0.317	(0.706); 0.049*
	SDHB ; male	0.081; 0.311	0.771; 0.056
	(SDHC) ; male	(0.221); 0.324	(0.630); 0.044*
	SDHD ; male	0.867; 0.398	< 0.001‡; 0.007†
	(SDHAF2) ; male	(0.253); 0.330	(0.528); 0.041*
	EPAS1 ; male	−0.175; 0.300	0.576; 0.067*
	NF1 ; male	0.298; 0.303	0.120; 0.056
	RET ; male	−0.948; 0.335	< 0.001‡; 0.026*
	(HRAS) ; male	−0.130; 0.313	0.694; 0.052
Transcriptional signature	cluster I; male	0.159; 0.323	0.332; 0.043*
Origin of mutations	germline; male	−0.014; 0.319	0.928; 0.047*
**COX-2 histologic pattern in tissue samples**			
Pearson correlation		*r*	*P* value
Mutation	*VHL*	0.040	0.700
	SDHx	0.266	0.009†
	(SDHA)	(−0.057)	(0.582)
	SDHB	0.092	0.375
	(SDHC)	(0.137)	(0.182)
	SDHD	0.194	0.058
	(SDHAF2)	(0.084)	(0.414)
	NF1	−0.031	0.767
	(HRAS)	(−0.081)	(0.431)
	(EPAS)	(−0.190)	(0.065)
Transcriptional signature	cluster I	0.197	0.054
Origin of mutations	germline	0.242	0.018*
