# Supplementary material for: Targeting Cyclooxygenase-2 in Pheochromocytoma and Paraganglioma: Focus on Genetic Background
Source: Cancers (Basel). 2019 May 28;11(6):743. doi: 10.3390/cancers11060743 (PMC6627450; doi:10.3390/cancers11060743)
Supplement: Supplementary file 1 [file cancers-11-00743-s001.zip › Table_S2_Pearson_correlation_COX-2_vs_hypxia-regulated_genes#02.pdf]

Table S2: Pearson correlation between expression of *COX-2* in the RNA sample cohort and 97 hypoxia-related genes in PPGLs

Hypoxia cross probes + SDH/VHL	Gene	*r*	*P* -value
A_24_P192485	TNFRSF11B	0.634	<0.001
A_23_P71530	TNFRSF11B	0.569	<0.001
A_23_P11685	PLA2G4A	0.564	<0.001
A_23_P34345	VCAM1	0.553	<0.001
A_23_P416395	STC2	0.553	<0.001
A_23_P17095	TFPI	0.541	<0.001
A_24_P96403	RUNX1	0.534	<0.001
A_24_P159434	CD300A	0.491	<0.001
A_24_P185854	DMD	0.487	<0.001
A_23_P202978	CASP1	0.485	<0.001
A_23_P121253	TNFSF10	0.482	<0.001
A_23_P58396	PDGFC	0.476	<0.001
A_23_P158725	SLC16A3	0.470	<0.001
A_23_P20494	NDRG1	0.470	<0.001
A_24_P48723	PTGIS	0.463	<0.001
A_23_P8108	HLA-DQB1	0.453	<0.001
A_24_P314179	ETS2	0.445	<0.001
A_23_P76992	PGF	0.432	<0.001
A_23_P257924	ETS2	0.429	<0.001
A_23_P17354	GDAP1L1	-0.420	<0.001
A_23_P150768	SLCO2B1	0.412	<0.001
A_24_P329795	C10orf10	0.396	<0.001
A_24_P393740	FYB	0.394	<0.001
A_23_P110686	STC2	0.392	<0.001
A_24_P148717	CCR1	0.391	<0.001
A_23_P330070	TFPI	0.390	<0.001
A_23_P91095	CD28	0.388	<0.001
A_24_P402222	HLA-DRB3	0.386	<0.001
A_23_P368691	ABCC9	0.377	<0.001
A_23_P207037	CD300A	0.374	<0.001
A_24_P935986	BCAT1	0.370	<0.001
A_23_P203920	SSPN	0.355	<0.001
A_23_P120472	TFAP2C	0.355	<0.001
A_24_P52921	BCAT1	0.353	<0.001
A_24_P125096	MT1X	0.351	<0.001
A_23_P303242	MT1X	0.350	<0.001
A_32_P175739	HK2	0.349	<0.001
A_23_P35597	C10orf10	0.345	<0.001
A_23_P165136	LRRC25	0.344	<0.001
A_24_P343695	RET	-0.339	<0.001
A_23_P151506	PLEK2	0.338	<0.001
A_23_P122127	FYB	0.337	<0.001
A_23_P113212	TMEM45A	0.331	<0.001
A_23_P137391	ENO1	0.331	<0.001
A_23_P29005	SAMSN1	0.331	<0.001
A_23_P327551	CPNE4	-0.327	<0.001
A_24_P365767	CYBB	0.320	0.001
A_23_P360379	EGLN3	0.318	0.001

A_23_P83579	ARNT2	-0.316	0.001
A_23_P87528	BCAT1	0.314	0.001
A_23_P85240	TLR7	0.309	0.002
A_24_P237586	ANKRD37	0.306	0.002
A_23_P571	SLC2A1	0.298	0.002
A_23_P145336	HLA-DRB3	0.295	0.003
A_24_P388528	ST6GAL1	0.286	0.004
A_32_P133072	SPON1	0.286	0.004
A_23_P315273	MT3	0.283	0.004
A_23_P136683	HLA-DQB1	0.279	0.005
A_23_P398460	HK2	0.269	0.006
A_23_P217258	CYBB	0.268	0.006
A_23_P38630	SSTR2	-0.266	0.007
A_23_P318800	SSTR2	-0.264	0.007
A_24_P111054	SLC2A5	-0.258	0.009
A_24_P382661	ETS2	0.254	0.010
A_23_P205355	SERPINA5	0.251	0.011
A_24_P10226	SEMA6D	-0.249	0.012
A_23_P51187	PRKCZ	0.245	0.013
A_23_P101208	CYB5A	0.244	0.013
A_23_P8175	PLAGL1	0.241	0.015
A_23_P129629	MT3	0.239	0.016
A_24_P300952	APLP2	-0.238	0.016
A_24_P321766	SERPINA5	0.236	0.017
A_23_P145846	MET	-0.232	0.019
A_24_P183994	RASEF	-0.231	0.020
A_24_P105501	ENO1	0.229	0.020
A_23_P65717	THSD4	-0.230	0.020
A_23_P90696	TRIB2	0.219	0.027
A_24_P404822	APLN	0.219	0.027
A_23_P407565	CX3CR1	0.218	0.028
A_23_P86012	LAMB3	0.218	0.028
A_23_P389281	HOXA13	-0.217	0.029
A_23_P201193	TSPAN2	0.211	0.033
A_23_P111311	AKAP12	0.207	0.037
A_23_P420442	SEMA6D	-0.200	0.044
A_24_P9090	HNRPDL	-0.197	0.047
A_24_P38387	NDRG1	0.195	0.050
A_23_P113393	APLN	0.191	0.055
A_24_P178503	ABCC9	0.189	0.057
A_23_P420692	PPFIA4	0.189	0.058
A_23_P145844	MET	-0.189	0.056
A_23_P338981	CYGB	-0.184	0.065
A_24_P67350	SLC24A3	-0.184	0.065
A_24_P305541	TRIB3	-0.179	0.072
A_32_P231568	RASEF	-0.173	0.082
A_23_P160159	SLC2A5	0.170	0.088
A_23_P325726	ACOT11	-0.169	0.090
A_23_P138635	BNIP3	0.167	0.093
A_23_P124084	LOXL1	0.167	0.094
A_23_P119943	IGFBP2	0.163	0.101
A_24_P295010	SERPINB9	0.163	0.101

A_23_P141346	MPP3	-0.163	0.101
A_24_P136866	SLC8A1	-0.160	0.107
A_23_P132027	SPAG4	0.156	0.118
A_32_P110372	SLC8A1	-0.152	0.128
A_24_P51037	C2orf3	-0.152	0.127
A_23_P214897	AKAP12	0.147	0.141
A_23_P31306	HOXA13	-0.144	0.149
A_23_P25674	CKB	-0.143	0.152
A_23_P321860	DMD	0.140	0.161
A_23_P124252	CAMK1D	0.138	0.166
A_24_P133288	PKP2	-0.138	0.167
A_23_P79978	SLC24A3	0.132	0.187
A_24_P5153	THSD4	-0.126	0.206
A_23_P91619	MIF	0.121	0.225
A_24_P309521	KCNJ5	-0.120	0.230
A_23_P91262	PTGIS	0.117	0.243
A_23_P218918	FGF2	-0.116	0.244
A_32_P208120	CAMK1D	0.115	0.250
A_23_P417415	ACOT11	-0.115	0.249
A_24_P917783	RUNX1	0.110	0.272
A_23_P141345	MPP3	-0.109	0.277
A_23_P201711	S100A6	0.108	0.281
A_24_P913900	SLC8A1	-0.105	0.293
A_24_P284353	TMEM107	0.104	0.300
A_24_P941441	GNA13	-0.101	0.311
A_24_P226755	TOX	0.098	0.328
A_23_P118791	TMEM107	0.097	0.333
A_23_P202939	APLP2	-0.097	0.333
A_23_P213153	HNRPDL	-0.097	0.331
A_32_P199796	DMD	-0.099	0.324
A_23_P202927	KCNJ5	-0.094	0.347
A_24_P34186	DMD	-0.085	0.397
A_23_P216167	PSD3	0.082	0.411
A_24_P163168	PDGFC	0.081	0.418
A_32_P228618	RBMS3	0.080	0.422
A_23_P78281	GNA13	-0.078	0.433
A_23_P162466	PKP2	-0.076	0.448
A_24_P62659	TSPAN2	0.074	0.461
A_24_P914519	CYBB	0.073	0.466
A_23_P356755	CEBPG	0.070	0.487
A_23_P117782	LARP6	-0.067	0.501
A_24_P924462	PRKCZ	-0.067	0.505
A_23_P210690	TRIB3	-0.063	0.528
A_23_P6849	CCR1	0.062	0.539
A_23_P128728	ARG2	0.059	0.554
A_32_P147078	SLC8A1	0.057	0.567
A_24_P61537	CKB	-0.058	0.565
A_23_P20392	PSD3	0.054	0.591
A_23_P52121	PDZK1	-0.053	0.600
A_23_P359245	MET	0.051	0.613
A_23_P10995	RBMS3	-0.049	0.621
A_24_P342388	DMD	0.048	0.630

A_24_P312041	PLAGL1	0.047	0.639
A_23_P26847	SOX9	0.047	0.640
A_24_P396753	TRIB2	0.044	0.660
A_23_P62437	TLR7	0.043	0.670
A_23_P30363	P4HA2	0.042	0.676
A_24_P390784	SREBF1	-0.042	0.677
A_23_P18966	P4HA2	-0.043	0.665
A_24_P239676	HLA-DQB1	0.040	0.688
A_23_P123413	TOX	0.040	0.692
A_32_P38323	SERPINB9	0.034	0.734
A_23_P129786	SREBF1	-0.032	0.752
A_23_P30687	SERPINB9	-0.030	0.761
A_24_P397043	ST6GAL1	-0.023	0.822
A_24_P18146	PSD3	0.020	0.844
A_23_P148249	THSD4	-0.016	0.869
A_24_P213134	SLC16A3	-0.015	0.878
A_23_P120062	C2orf3	-0.014	0.890
A_24_P359942	RBMS3	-0.012	0.908
A_23_P147755	APLN	0.000	0.997
