# Supplementary figures and images for: Targeting Cyclooxygenase-2 in Pheochromocytoma and Paraganglioma: Focus on Genetic Background
Source: Cancers (Basel). 2019 May 28;11(6):743. doi: 10.3390/cancers11060743 (PMC6627450; doi:10.3390/cancers11060743)

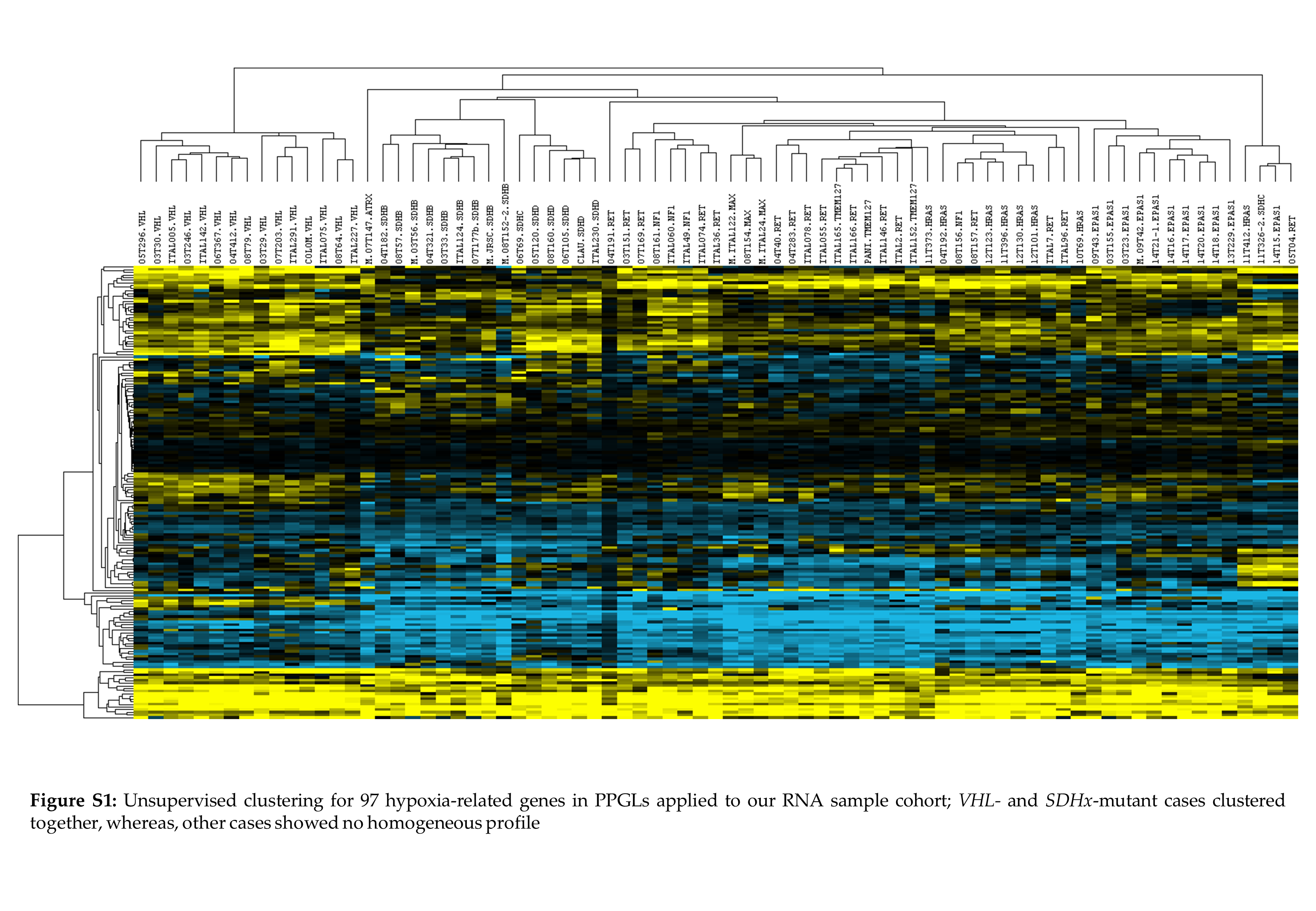

Supplement: Supplementary file 1 [file cancers-11-00743-s001.zip › Figure_S1-Clustering_hypoxia-related_genes.tif]
